# Mouse chromocenters DNA content: sequencing and in silico analysis

**DOI:** 10.1186/s12864-018-4534-z

**Published:** 2018-02-20

**Authors:** Dmitrii I. Ostromyshenskii, Ekaterina N. Chernyaeva, Inna S. Kuznetsova, Olga I. Podgornaya

**Affiliations:** 1 Institute of Cytology RAS, St.-Petersburg, 194064 Russia; 2 Far Eastern Federal University, Vladivostok, 690922 Russia; 3 St Petersburg State University, St Petersburg, 199034 Russia; 4 School of Biomedical Sciences, The Chinese University of Hong Kong, Shatin, Hong Kong

## Abstract

**Background:**

Chromocenters are defined as a punctate condensed blocks of chromatin in the interphase cell nuclei of certain cell types with unknown biological significance. In recent years a progress in revealing of chromocenters protein content has been made although the details of DNA content within constitutive heterochromatin still remain unclear. It is known that these regions are enriched in tandem repeats (TR) and transposable elements. Quick improvement of genome sequencing does not help to assemble the heterochromatic regions due to lack of appropriate bioinformatics techniques.

**Results:**

Chromocenters DNA have been isolated by a biochemical approach from mouse liver cells nuclei and sequenced on the Illumina MiSeq resulting in ChrmC dataset. Analysis of ChrmC dataset by the bioinformatics tools available revealed that the major component of chromocenter DNA are TRs: ~ 66% MaSat and ~ 4% MiSat. Other previously classified TR families constitute ~ 1% of ChrmC dataset. About 6% of chromocenters DNA are mostly unannotated sequences. In the contigs assembled with IDBA_UD there are many fragments of heterochromatic Y-chromosome, rDNA and other pseudo-genes and non-coding DNA. A protein coding *sfi1* homolog gene fragment was also found in contigs. The S*fi1* homolog gene is located on the chromosome 11 in the reference genome very close to the Golden Pass Gap (a ~ 3 Mb empty region reserved to the pericentromeric region) and proves the purity of chromocenters isolation. The second major fraction are non-LTR retroposons (SINE and LINE) with overwhelming majority of LINE - ~ 11% of ChrmC. Most of the LINE fragments are from the ~ 2 kb region at the end of the 2nd ORF and its’ flanking region. The precise LINEs’ segment of ~ 2 kb is the necessary mouse constitutive heterohromatin component together with TR. The third most abundant fraction are ERVs. The ERV distribution in chromocenters differs from the whole genome: IAP (ERV2 class) is the most numerous in ChrmC while MaLR (ERV3 class) prevails in the reference genome. IAP and its LTR also prevail in TR containing contigs extracted from the WGS dataset. In silico prediction of IAP and LINE fragments in chromocenters was confirmed by direct fluorescent in situ hybridization (FISH).

**Conclusion:**

Our data of chromocenters’ DNA (ChrmC) sequencing demonstrate that IAP with LTR and a precise ~ 2 kb fragment of LINE represent a substantial fraction of mouse chromocenters (constitutive heteroсhromatin) along with TRs.

**Electronic supplementary material:**

The online version of this article (10.1186/s12864-018-4534-z) contains supplementary material, which is available to authorized users.

## Background

Repetitive DNA sequences may account for more than two thirds of the mammalian genomes [1], yet their regulatory and architectural role remains largely enigmatic, partly because it is difficult to study them with molecular biology techniques. Main part of the repetitive DNA does not encode any proteins being truncated and thus regarded as noncoding DNA. There is a growing body of evidence that noncoding DNA is essential for regulation of complex spatiotemporal gene expression patterns in different mammalian species [2, 3]. Repetitive DNA tends to form a densely staining aggregation of heterochromatic regions in the nucleus called chromocenters [4]. Chromocenters are defined as a punctate condensed collection of chromatin in the interphase cell nuclei of curtain cell types with unknown biological significance [5]. Complexes of specific proteins together with pericentromeric (periCEN) and centromeric (CEN) satellite repeats are condensed into constitutive heterochromatin and produce cytologically visible chromocenters in the interphase nuclei. Chromocenters are considered to comprise a repressive environment in the nucleus [6, 7].

The role of chromocenters in the nuclear architecture and arrangements of chromosome territories in the nuclear space is widely suggested. During neuronal maturation, the nuclear morphology of the neuron changes from a small, heterochromatic nucleus with many randomly-located chromocenters and nucleoli, to a large, mostly euchromatic nucleus with fewer, larger chromocenters associated with a large, centrally located nucleolus [8–12]. This non-random reorganization suggests that these changes occur via clustering and relocation of these structures during terminal differentiation and these global chromatin changes have been observed in terminally differentiating neurons in a variety of species, strongly indicating functional significance [9, 13]. Association of the human artificial chromosomes (HACs) with chromocenters is crucial for their stability in mouse cells, i.e. these experiments suggest the ability of chromocenters to fix inserted DNA [14]. The investigation of the spatial intranuclear arrangement of HACs in a xenospecific mouse background by using FISH and 4C–seq technologies shows that the chromatin segments acquire respective positions in the nucleus suggesting that this is their intrinsic property. Results of several studies suggest that building of a functional nucleus is largely a self-organizing process based on mutual recognition of chromosome segments belonging to the major chromatin classes defined according to tandem repeats (TR), LINE and SINE enrichment [15]. The chromocenter acts as a hub for the deposition of heterochromatic markers, controlling CEN/periCEN DNA replication timing and chromosome segregation. Murine periCEN major satellite (MaSat) is highly transcribed during embryogenesis, and transcripts are responsible for reorganization of periCEN DNA into chromocenters. Destruction of these transcripts led to developmental arrest indicating their role in de novo heterochromatin formation and proper developmental progression [16]. It appears that heterochromatin has so far revealed only a very small part of its secret message. More thorough characterization of CEN/periCEN RNA structure and function will be an important challenge for the future. Investigation of the transcription burst may prove to be vital in resolving at least some of the mysteries surrounding the role of constitutive heterochromatin in development, cell differentiation and responses to stress, although RNA only could be recognized if corresponding DNA sequences are known.

In recent years progress in revealing chromocenter protein content has been achieved [17, 18] but it is still not clear what DNA underlay constitutive heterochromain. Enrichment in tandem repeats and many transposable elements (TE) are characteristic for chromocenters [6]. Although historically relegated as “junk DNA”, tandem repeats (TR) have taken on a new importance with the realization that their tandem organization provides potentially unique functional characteristics. Tandemly repeated DNA is organized as multiple copies of a homologous DNA sequence of a certain size (repeat unit or monomer) that are arranged in a head to tail pattern to form tandem arrays, and thus represents a distinct type of sequence organization shared by all higher eukaryotes sequenced genomes [19, 20]. The enrichment of CEN and periCEN regions with TR in all organisms from fission yeast to humans appearing to be critically important for establishing heterochromatin formation and proper chromosome segregation [21].

Recent progress in genome sequencing techniques does not help to assemble the heterochromatic regions due to their enrichment with the TR. Such regions could not be assembled by current bioinformatics methods [22, 23]. The overwhelming majority of the assembled genomes contain a 3 Mb (Golden Path Gap, GPG) empty region around each CEN. The appearance of the Single molecule real time sequencing (SMRT, PacBio) technique seems to be promising for TR rich regions assembly, but it is still in progress [24]. The repetitive DNA rich regions assembly remains a challenge even with the new generation sequencing due to the poor knowledge about repetitive DNA, especially TR, itself. The composition of chromocenters has not been determined up to now [6]. We used a previously published biochemical approach [25] in order to determine a chromocenter composition with the bioinformatics tools available. Due to the fact that whole-genome sequencing studies of eukaryotic organisms have focused solely on euchromatic regions, the tools for the repetitive DNA enriched regions analysis are quite limited.

It was determined in pre-genomic era, that CEN and periCEN regions of the house mouse, *Mus musculus*, contain two highly conserved, tandemly repeated sequences known as minor and major satellites (MiSat and MaSat, respectively, SATMIN and GSAT_MM in Repbase nomenclature). The MaSat is composed of 234-bp monomers and is located in the region flanking the CEN whereas MiSat consists of a 120 bp monomer unit and is present at the CEN of each chromosome except Y. MaSat field of 300–600 kb occupies the terminal region of all mouse telocentric (single-armed) chromosomes and abut to the MiSat field, which serves as the site of kinetochore formation and spindle microtubule attachment [26–29]. MiSat and MaSat are routinely used to mark mouse CEN or periCEN regions [30–33].

Genome wide analysis of the large TR found in the mouse genome has been done. Large tandem repeat (TR) was defined as (1) a genomic fragment with monomers tandemly arranged without inserts, (2) the monomer length was set to be less than 2 kb, (3) the monomer array length was set to be over 3 kb. Such a definition allowed to find in the databases TR with maximal similarity to the “classical” satDNA with mini- and micro-satellites left behind [34].

Even with mouse TR classified, the whole chromocenter DNA content remains to be unknown while the content of surrounding area, which is also involved in heterochromatinisation, deserves determination [7]. It became clear that heterochromatic compartments, i.e. chromocenters, are quite complicated structures far of being uniform. Not only TR of different families but precise LINE fragments are involved in their formation [35].

The lack of bioinformatics techniques forced us to combine possible approaches in order to determine an approximate chromocenter content. The results of the current work gave a number of sequences, which could clarify the complex chromocenter content and composition. Besides, our data help to increase the collection of the murine heterochromatic probes, which is especially important for such an extensively used model as the laboratory mouse, a subject for molecular biology researches, accomplishing complex methodologies including genome sequencing. We made an attempt to describe DNA composition of the mouse heterochromatic regions. We applied High-Throughput sequencing (HTS) of DNA from biochemically isolated chromocenters for further analyses and as a result obtained a representative view of murine chromocenters DNA content.

## Methods

### Animals and chromocenter isolation

The *Mus musculus* line has been kept in the Institute of Cytology RAS (INC RAS, St. Petersburg, Russia) according to the approved standards in the Laboratory Animal Resources facility at INC RAS animal house. Chromocenters have been isolated using biochemical approach from nuclei of mouse liver (laboratory strain 129P2, 4 to 6 weeks old, weight ~ 20 g) by high centrifugal force through gradients of sucrose according to the method published [25, 36]. Method is based on the higher resistance of chromocenters to low ionic strength treatment as compared with that of nucleoli and euchromatin. The method allows separation of chromocenters that are essentially free of nucleoli and other nuclear contaminants [25].

### Library construction, sequencing annotation

Sequencing library was prepared using the Nextera DNA Sample Preparation Kit (Illumina, USA). The median insert size was about 100 bp. Libraries were sequenced on the Illumina MiSeq System. 4,371,191 non-overlaped paired-end reads with length 37 bp were generated. For repeat content analysis we combined paired-end reads sets in united dataset and resulting set of 8,742,382 reads was counted as single paired.

The quality of raw reads in fastq format was assessed using the FASTQC [37] program. Raw reads were cleaned and filtered with trimmomatic program [38] that removed all reads containing Illumina technical sequences, reads with average phred (nucleotide base call) score low 25 in window 4 bp and reads shorter than 30 bp. Dataset was submitted to SRA database with SRP073677 accession number. After cleaning number of reads was reduced by 21.6% and contains 6,854,028 non-paired reads. Around 98% of these reads could be mapped to the mouse reference genome and contigs unplaced. To determine the number of reads mapped, Bowtie2 [39] was used with the -local-sensitive on pre-built Bowtie2 index for the GRCm38 genome version. The unmapped reads were discarded from subsequent analysis. The dispersed elements of different classes (LINE, ERV и DNA transposons) extracted from the genome version GRCm38.p6, according to their coordinates in RepeatMasker outfile (ftp://ftp.ncbi.nlm.nih.gov/genomes/all/GCF/000/001/635/GCF_000001635.26_GRCm38.p6/GCF_000001635.26_GRCm38.p6_rm.out). The TR content was established on the base of TR classification published [34]. Chromocenters reads were mapped to the repeats from genome (Table 1) and TR arrays (Tables 1 and 4) using Bowtie2 [39] with --local-sensitive parameter.

**Table 1:** The relative amount of different repeat classes in the reads’ set estimated by comparison with reads mapping (Bowtie2)

			ChrmC %	wgHTS %	Reference genome %
ERV	All		8.8	12.7	12.3
	ERV3		3.2	5.9	6.1
	ERV2	All	5.1	5.9	5
		IAP	2.2	0.8	1
	ERV1		0.5	0.9	1.2
LINE			10.9	17.1	19.9
SINE			2.1	5.2	8.3
DNA transposons			0.6	1.2	1.2
TR		MaSat	66.2	13.0	*
		MiSat	4.4	0.6	*
		Other	0.9	0.9	*
		Tel**	0.1	0.1<	*
All			93.7	50.8	–

* - poorly annotated in the reference genome, content < 0,1%; ** - telomeres, the actual amount in wgHTS is 0.04%; the figures up to the second decimal place shown in the table

We used whole genome High-throughput sequencing (wgHTS) of the house mouse 129P2 strain on Illumina Genome Analizer II (we used run with Sequence Read Archive (https://trace.ncbi.nlm.nih.gov/Traces/sra) accession numbers ERR007731, ERR007732, ERR007733, ERR007771 from study with Sequence Read Archive accession numbers ERP000034) to evaluate the repeat content in the wgHTS data. In the genome assembly the amount of each class was counted in the following way: the length of the each class (LINE, SINE, ERV etc) fragments was summarized and the resulting sum was divided by GRCm38.p6 assembly length as denominator (Table 1).

**Fig. 1:**
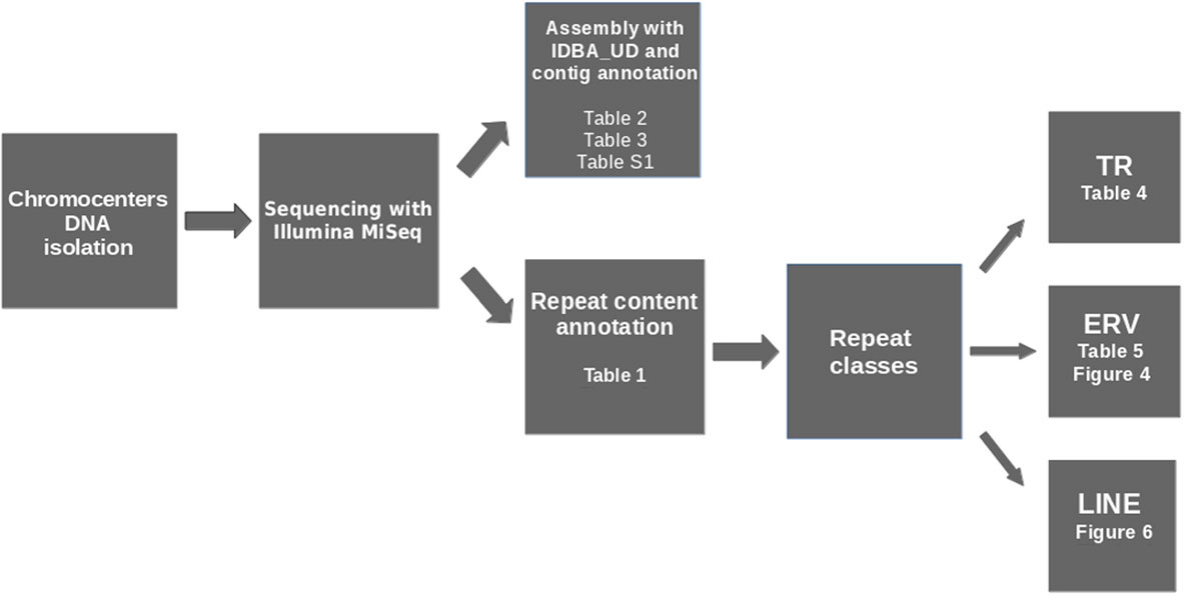
Chromocenters’ DNA sequencing and resulting reads analysis. Table or figure on which the results of each step are given, indicated

The scheme of the work with the reference to the following tables and figures is given on Fig. 1.

### Contig assembly and annotation

We used raw paired-end reads cleaned and filtered as mentioned above for contig assembly. All reads were mapped to duplicated MaSat and MiSat consensus sequences obtained from Repbase database (GSAT_MM and SATMIN) with Bowtie2 with --very-sensitive-local flag. Successfully mapped reads were separated from main dataset. These reads omitted from the set and the reads remained were assembled with IDBA_UD assembler with default parameters [40]. Assembly contigs (Additional file 1: Table S1) were compared to the mouse reference genome (ver. GRCm38.p6) and Repbase collection to get annotation. All sequence comparisons were performed using standard algorithms such as BLAST [41]. To check for the presence of repeat elements the sequence sets were searched against the Repbase database [42] using CENSOR (Ver: 4.2.28) with follow parameters: default, nofilter, minsim 0.75, show_simple, bprg blastn, mode norm.

### Search of TR and ERV fragments in mouse WGS database

We have searched throw mouse Whole Genome sequencing (WGS) project AAHY01 (https://www.ncbi.nlm.nih.gov/Traces/wgs/?val=AAHY01) for the contigs, which contain TR (MaSat, Misat, TRPC-21A [34]) together with fragments of ERV. WGS contigs with at least one monomer of MaSat or MiSat or three monomers of TRPC-21A and ERV fragment of length > 400 bp (LTR) determined by BLAST, considered as positive. We searched for ERV internal fragment and LTR from Repbase in these contigs. The hits with e-value >1e-10 and the score < 100 was discarded.

### Chromosomes preparation

3–6 month-old CBA mice were housed and maintained according to the approved standards in the Laboratory Animal Resources facility at Institute of Cytology RAS (St. Petersburg, Russia). Metaphase chromosome in suspensions were isolated from mouse bone morrow and L929 cell line according to a standard cytogenetic protocol [43]. Chromosome suspensions were fixed in methanol:acetic acid (3:1).

### Fluorescence in situ hybridization (FISH) and fiberFISH

The following DNA fragments were used as probes for FISH: the MiSat fragment (362 bp) inserted in pGEM7 vector [31]; 5′, 3’ Cy3-dUTP labelled synthetic single stranded DNA IAP probe 196 bp (Sintol, Moscow, Russia; Additional file 2: Supplementary 1). MaSat and MiSat FISH probes were prepared by PCR amplification using digoxigenin-11-dUTP (Roche) or biotin-16-dUTP (Roche). FISH was conducted according to previously published protocol with a few modifications [44]. In brief, FISH probes mixture (50% formamide, 10% dextran sulfate, 50 ng of each labeled DNA in 2× SSC) was applied to the slide, covered with a coverslip, and sealed with rubber cement. The slide was then denatured for 2 min at 80 °C and incubated at 37 °C overnight. After hybridization the slide were washed according standard FISH protocol. FITC-conjugated anti-DIG antibody (Roche) and Streptavidin, Alexa Fluor 594 conjugate were used for detection digoxigenin and biotin labeled probes accordingly. Finally, the slides were counterstained with DAPI (4 ′, 6-diami-dino-2-phenylindole) and mounted in an antifade solution (Vectashield, Vector laboratories, Burlingame, CA, USA).

Fiber-FISH with IAP and MiSat probes was conducted according to previously published protocol with a few modifications [44]. Primary fibroblast cells were lysis in buffer (0.5%SDS, 5 mM EDTA, 100 mM Tris, pH 7.0). Then slowly drag the solution down the slide. Slides were air dry and fix ethanol:acetic acid (3:1). DNA-fiber slides were hybridized with DIG-labelled MiSat and cy3-labelled IAP probes as described above.

**Synthetic IAP** probe of 196 bp was based on consensus sequence of IAP fragments from 5 mouse BAC-clone (Additional file 2: Supplementary 1). IAP probe shows the similarity up to 95% with IAP in BAC clones. Synthetic IAP fragment shows maximum similarity with the Repbase consensus of IAP-d with deletion and the similarity with it is no more than 70%.

**Graphic data visualization** were performed with Pytnon’s matplotlib library (www.matplotlib.org).

## Results

Sequencing library prepared by the Nextera kit was sequenced on the Illumina MiSeq Platform. The approximate number of 37 bp reads after cleaning and filtering was ~ 6.5 millions. Thus, the resulting sequencing output was ~ 250 Mb, which corresponds to 2.5× coverage of the whole GPG in mouse genome. The set of reads was used for the repetitive DNA content analysis, quantification and assembly. This set is referred to here as “ChrmC”.

### Repetitive DNA quantification

The repeats represent about 94% of chromocenters’ DNA (ChrmC). Tandem repeats (TR) are the major representative: ~ 71% in total, including ~ 66% of MaSat and ~ 4% of MiSat. The second most abundant class is Non-LTR retroposons (SINEs and LINEs) with overwhelming majority of LINEs - ~ 11%. The third most abundant (~ 9%) are ERVs (endogeneous retroviruses). Less than 1% represent DNA transposons (Table 1). The well known TR underrepresentation in sequencing data and the difficulties in assembling [24, 34] is visible from the data comparison. The underrepresentation of SINE in ChrmC is visible in comparison with wgHTS and especially with the reference (assembled) genome. The ERV different classes asymmetry distribution could be expected (Table 1).

MaSat- and MiSat-containing reads were discarded from the set of ChrmC DNA and the rest was assembled with IDBA_UD with future annotation.

### Contig assembly and annotation

Reads without MaSat and MiSat were assembled with IDBA_UD with default parameters. IDBA_UD produced 93 contigs > 300 bp with N50 equal to 643 bp with maximal contigs’ length 4385 bp, mean contig length 616 bp, and total assembly length 57,286 bp (Additional file 1: Table S1). ERV are the most abundant family of transposable elements in the contigs assembled. Thirty five out of 93 contigs of chromocenters assembly with total length of ~ 26 kbp contain ERV fragments. Fragments of all three ERV classes (1–3) represented in Repbase are found in the contigs of chromocenters assembly; ERV2 (including IAP) is prevailing. In addition to ERVs’ fragments, one of contigs contained SINE fragments; two contigs were the LINE fragments; four contigs were composed of the fragments of rDNA pseudogenes (Table 2).

**Table 2:** Repeats fragment found in assembled contigs

Family	Subfamily	Fragments	Length
Endogenous Retrovirus			
	ERV	7	3384
	ERV1	6	3300
	ERV2	20	8745
	ERV3	10	7107
Non-LTR Retrotransposon			
	L1	2	681
	SINE2/tRNA	1	194
Pseudogene			
	rDNA	4	3587
Total		50	26,998

Endogenous retroviruses are the most abundant family of transposable elements in assembled contigs. Column names means: fragments – the amount of contigs’ fragments with similarity to the subfamily indicated; length – total fragments’ length of the subfamily indicated

Fifty contigs, which did not contain fragments of the repetitive sequences from Repbase were annotated by BLAST against the reference mouse genome. The major part of them (31) turned out to be unannotated dispersed sex chromosome sequences. Among them 19 contigs were found only on the Y chromosome, where they are repeated more than 500 times. 12 remaining contigs were found both on Y and X chromosomes. These sequences were repeated more than 500 times on the Y chromosome and only 10–20 times on the X chromosome (Table 3).

**Table 3:** Contigs without similarity to Repbase annotated against reference genome

Chromosome	Chromoband	Annotation	Number of contigs
various	various	rDNA	8
7	B5	The Prader-Willi syndrome region (Ipw)	6
11	A1	Sfi1 homolog	1
13	B3	V2R pseudogene locus	2
14	A1	unannotated	2
X,Y	dispersed	unannotated	12
Y	dispersed	unannotated	19
		Total	50

The number of contigs, their chromosome position and annotation is given

Ribosomal DNA (rDNA) was found in 8 contigs. Six contigs were annotated as members of the imprinted gene in the Prader-Willi syndrome region from chromosome 7 (Pwcr1), whereas all of them are present in multiple copies within this region (> 100 hits). Genomic sequence analysis of Pwcr1 confirmed the presence of multiple copies that are organized within local tandem-repeat clusters [45].

One contig represents a fragment of S*fi1* homolog gene. It is localized in the chromosome 11 periCEN region. *Sfi1* is known to play a role in the dynamic structure of centrosome-associated contractile fibers via interaction with CETN2 (centrin2), centrosomes’ conserved calcium-binding proteins unique to eukaryotes [46, 47]. The close proximity of this gene to GPG explains its presence in the chromocenter specific library.

Two contigs align as multiple copies to the V2R pseudogene cluster on chromosome 13 [48]. Two more contigs align as multiple copies (hits > 200) to the region of 0.1–4.5 Mb from GPG of chromosome 14 (Table 3).

The list of contigs, annotation and Genbank ID are given in Additional file 1: Table S1. The overview of the annotated contigs shows that the main part of the fragments are repetitive sequences in the reference genome.

### Tandem repeats (TR) in ChrmC dataset

In a previously published classification [34] only TRs with the monomer length < 2 kb and TR arrays’ length > 3 kb were taken into consideration. The TR estimation in ChrmC reads set was accomplished by comparison with the known TR arrays of the mouse genome (Table 4).

**Table 4:**
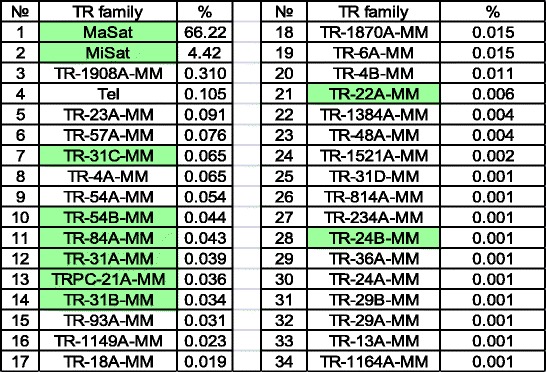
Percentage of reads mapped to TR (tandem repeat) array with Bowtie2 tool

TR from mouse WGS database in descending order of their amount. TRs tested as CEN/periCEN are colored by green. Tel - telomeric repeats in ChrmC

The overwhelming majority was still MaSat (~ 66%) and the second abundant was MiSat (~ 4%). The rest of TR families constitute ~ 1%. Some TRs have been already tested as chromocenter-associated (colored by green in Table 4; Additional file 3: Figure S1; [34, 49]). Well represented TRs with long monomers (> 1 kb) did not have any similarity with Repbase consensuses, so they are not derived from TE present in Repbase.

Telomeric DNA of most eukaryotes is composed of short tandemly repeated sequences T_2_AG_3_ for all vertebrates and thus it represents a TR class [31, 50]*.* Telomeric repeat is absent in Repbase, so the search for it was accomplished by mapping of ChrmC dataset to (T_2_AG_3_)_20_. It was revealed in a reasonable amount (~ 0.1%, Table 4) which is in agreement with previously published data [51].

### ERV in chromocenters

**Fig. 2:**
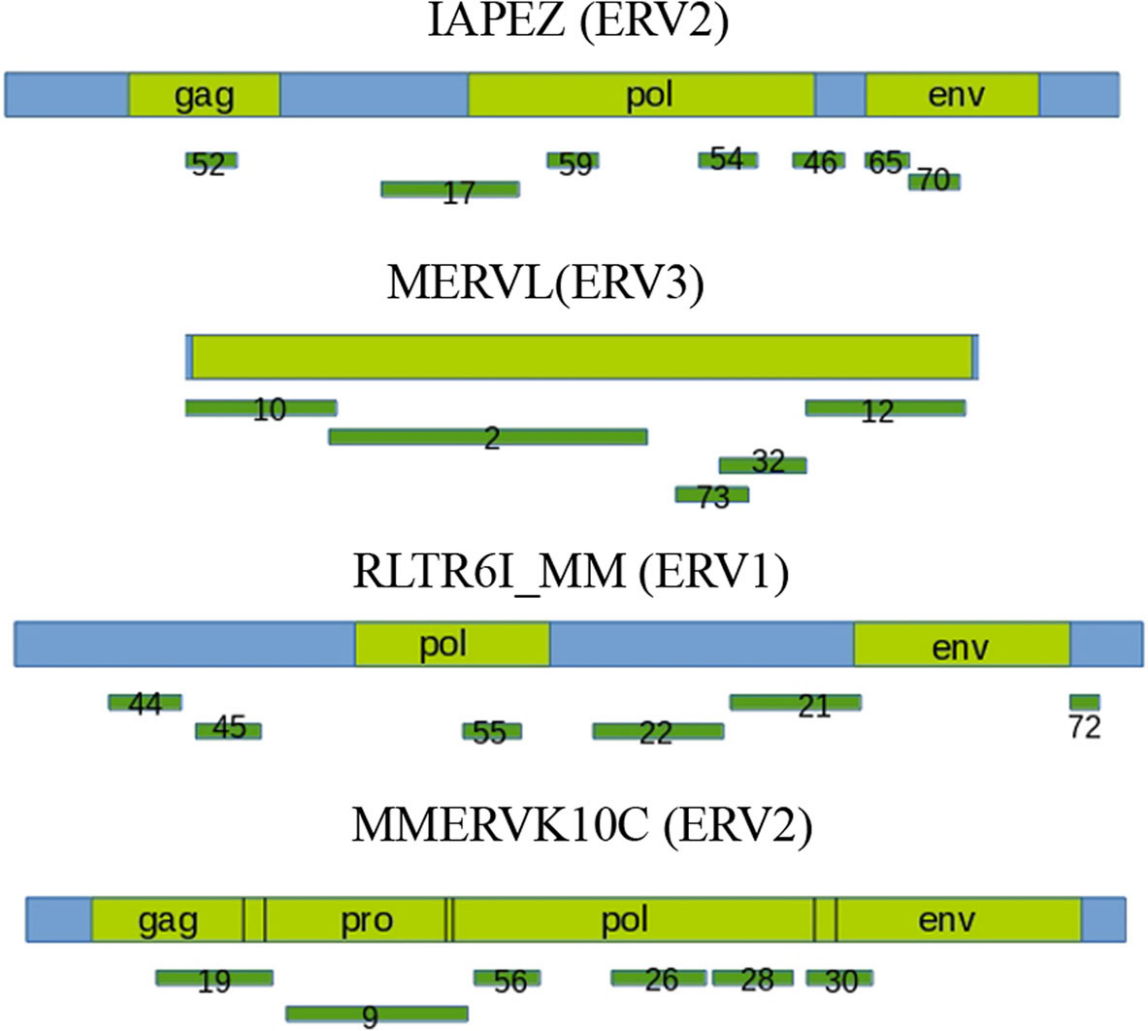
Mapping of assembled contigs on ERVs consensus sequence. Number of contig mapped is shown according to Additional file 1: Table S1. Position of *gag, pro, pol* and *env* genes inside ERV are shown in green. Blue – non-ORF internal ERV sequences

Vertebrate-specific endogenous retroviruses (ERV) represent a superfamily of murine LTR retrotransposons. Most ERVs have a high degree of homology to each other and to modern exogenous retroviruses and this was the basis of their classification [52]. Despite their truncation in the genome, the remaining viral ORFs (e.g. *gag*, *pro*, *pol*, and *env*) could be recognized and used for a tuned classification (Fig. 2).

ERV were the third most abundant representatives in ChrmC DNA (Table 1) but the first most abundant in assembled contigs (Table 2). In assembled contigs 42 fragments of 35 contigs belong to the ERV class (Table 2). They aligned to different ERV consensuses from the Repbase (Fig. 2). In contrast to TRs, where most monomers are short (Table 4), ERVs are rather long sequences – about 6 kb according to Repbase. It was interesting to determine the form of ERV existence in ChrmC - whether the full sized ERVs or only their fragments are present in the constitutive heterochromatin.

Contigs mapping to the different classes of ERV from Repbase ERV internal sequences ERV1 (RLTR6I_MM), ERV2 (IAPEZI, MMERVK10C) and ERV3 (MERVL) disclosed that contigs cover the whole consensus (ERV3 (MERVL)) or with little gaps (Fig. 2).

**Fig. 3:**
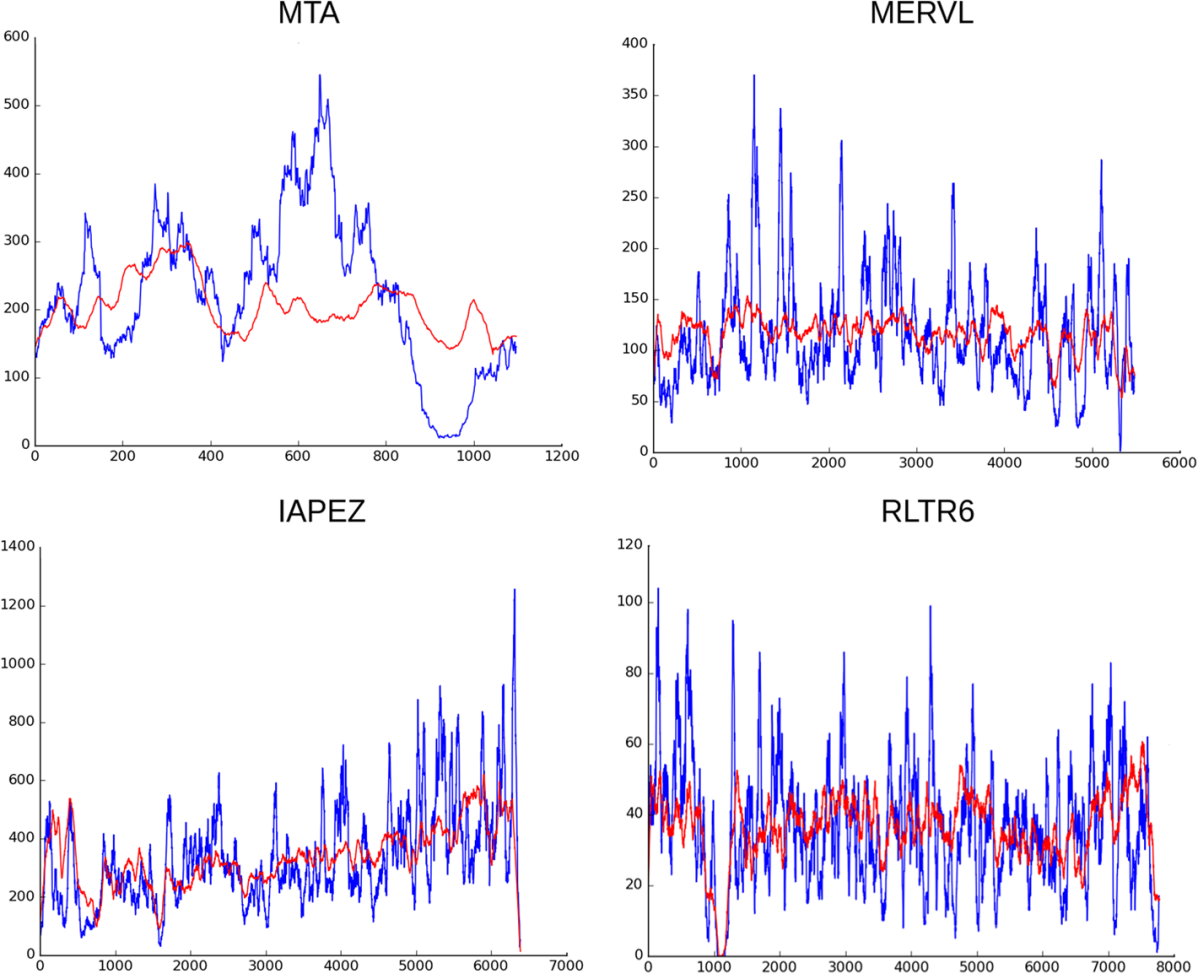
Reads coverage on mouse ERVs internal part consensus from Repbase. Blue line – reads from ChrmC. Red line – reads from wgHTS normalized on dataset size. X axis – consensus of ERV indicated in bp; Y axis – each read was mapped to the consensus and the coverage of each nucleotide indicated is shown

We used ChrmC reads together with wgHTS in order to check their coverage with ERV consensus sequences from Repbase (Fig. 3). MTA is a specific sequence for all rodents; ~ 1 kb MTA element is the most ancient and most truncated. MTA belongs to the MaLR-LTR (Mammalian apparent LTR-retrotransposons) group. MTA transposons have structural similarities to ERV3, and are related to human THE1 [53]. There was no enrichment of any ChrmC fragments covering MTA Repbase consensus (Fig. 3), which may imply that the whole MTA copies present in chromocenters. The seeming difference of MTA graph from the rest may be explained by the difference in the scale for MTA, as it is the shortest among the other ERVs.

For IAPEZI, MERV3, RTLR6I_MM some peaks visible on graphs (Fig. 3) could result from the real sequence variability in comparison to Repbase consensus used as the blueprint. The coverage happens to be rather uniform for all consensuses (Fig. 3), which demonstrates that no partial fragments of ERV are overrepresented.

The amount of reads similar to the main ERV consensus sequences in the whole set of reads in ChrmC DNA was counted by Bowtie2 (Table 5). Three first lines of the “internal part” are occupied by IAPEZI, MERVL and rodent-specific MTA.

**Table 5:** Percentage of ChrmC reads mapped to ERV internal sequences and LTRs with Bowtie2 tool

ERV internal part				ERV LTR			
	Repbase name	Read mapped %	Class		Repbase name	Read mapped%	Class
1	IAPEz-int	1.148	ERV2	1	MTA_Mm	0.274	ERV3
2	MERV3-int	0.238	ERV3	2	MTD	0.260	ERV3
3	MTA_Mm-int	0.164	ERV3	3	MT2B	0.241	ERV3
4	IAPEY3-int	0.163	ERV2	4	MTC	0.223	ERV3
5	RLTR6-int	0.131	ERV2	5	RLTR12B	0.221	ERV2
6	ORR1B1-int	0.122	ERV3	6	RLTR20A4	0.201	ERV2
7	MMERVK10C-int	0.119	ERV2	7	RLTR9D	0.176	ERV2
8	RLTR10-int	0.153	ERV3	8	RMER20A	0.141	ERV2
9	MTD-int	0.108	ERV2	9	MTEb	0.114	ERV3
10	ORR1D1-int	0.093	ERV2	10	MTB	0.110	ERV3

The sequence names are according to Repbase. Ten most numerous elements are shown

### ERV representation in TR containing contigs and IAP probe

We checked the ERVs in the mouse WGS dataset. Both ERVs’ LTR and internal parts are under consideration. The contigs, which contain TR and ERV fragments together were separated from the mouse WGS dataset. Contigs with MaSat, MiSat and TRPC-21A were selected as these TRs are certainly members of CEN/periCEN region.

**Fig. 4:**
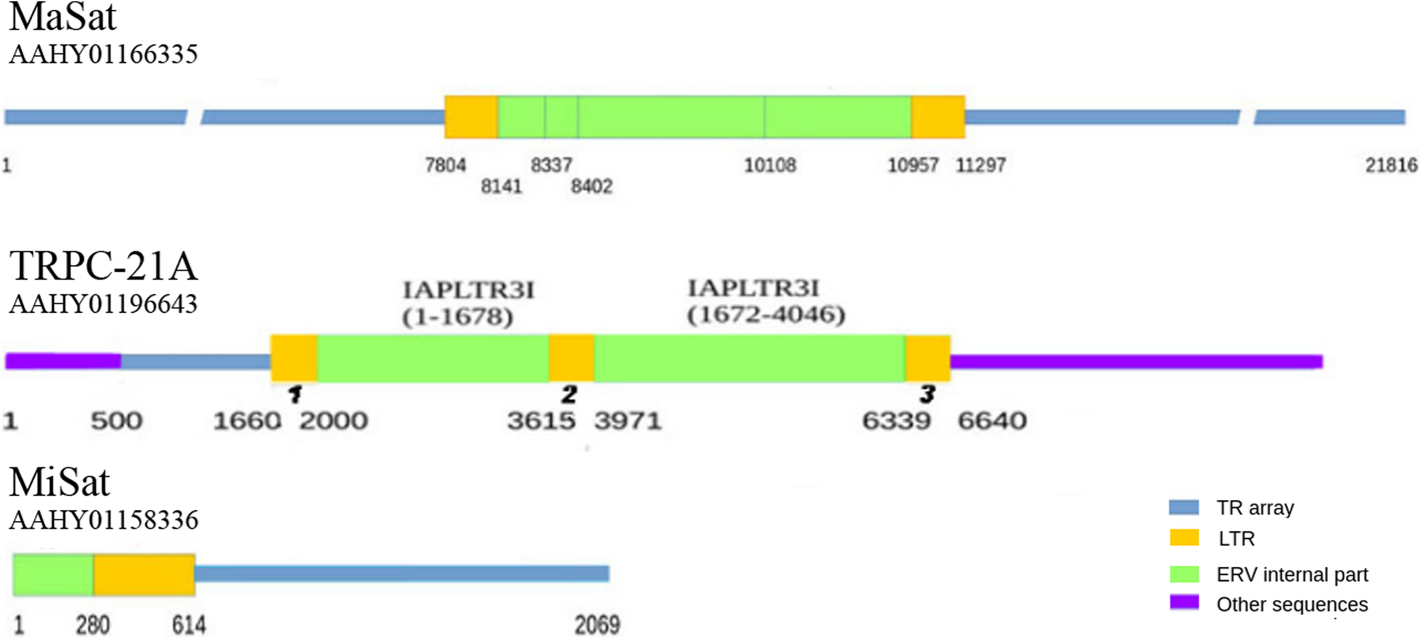
Examples of typical TR containing contigs found in the WGS database. The contig ID is under the TR name used in search. Color legend is at the right bottom. IAP I is the IAP internal part. Figures under the line represent nucleotide number from the beginning of each contig. At the panel TRPC-21A miscellaneous retroposon fragments marked by purple line; numbers in bold italic: *1 –* IAPLTR3*, 2 –* IAPY2_LTR*, 3* – IAPLTR3

About 2000 contigs with MaSat and ERV fragments were identified; the resulting tables for MiSat and TRPC-21A contain 34 and 29 contigs (Tables S2 and S3). The amount of contigs found poorly reflect TR representation in the genome but rather the success of WGS contigs assembly. The MaSat is the mostly composite TR, so it produces 751 arrays with the maximum length of ~ 23 kb, while TRPC-21A and MiSat are more homogeneous with 50 and 21 arrays and maximum length ~ 33 kb and ~ 6 kb, respectively [34]. The MiSat homogeneity leads to the lack of reference points, which resulted in very short contigs due to the poor assembly of MiSat arrays (Fig. 4, MiSat). In the current search, the length of the array was not considered if ERV presence was detected.

No less than half of TR contacting ERV fragments was represent IAP (Tables S2 and S3). No full-length IAP and other ERV were found in the contigs. The vast majority of contigs contained an ERV internal fragment together with at least one LTR. The most characteristic picture of the distribution in contigs is that the internal part of one IAP (Fig. 4, TRPC-21A) or mixture of the internal fragments of different IAP types (Fig. 4, MaSat) surrounded by the LTRs in ordinary orientation and with ordinary length. Thus, all the fragments from ChrmC contigs could be found also in WGS; internal fragments were often mixed but the typical IAP (or other ERV) structure with internal part and LTR at the borders remained.

The in silico prediction of IAP being the main ERV heterochromatic component should be proved in situ, so the probe for IAP (ERV2) developed based on the alignment of fragments found in several contigs mouse BACs (Additional file 2: Supplementary 1).

**Fig. 5:**
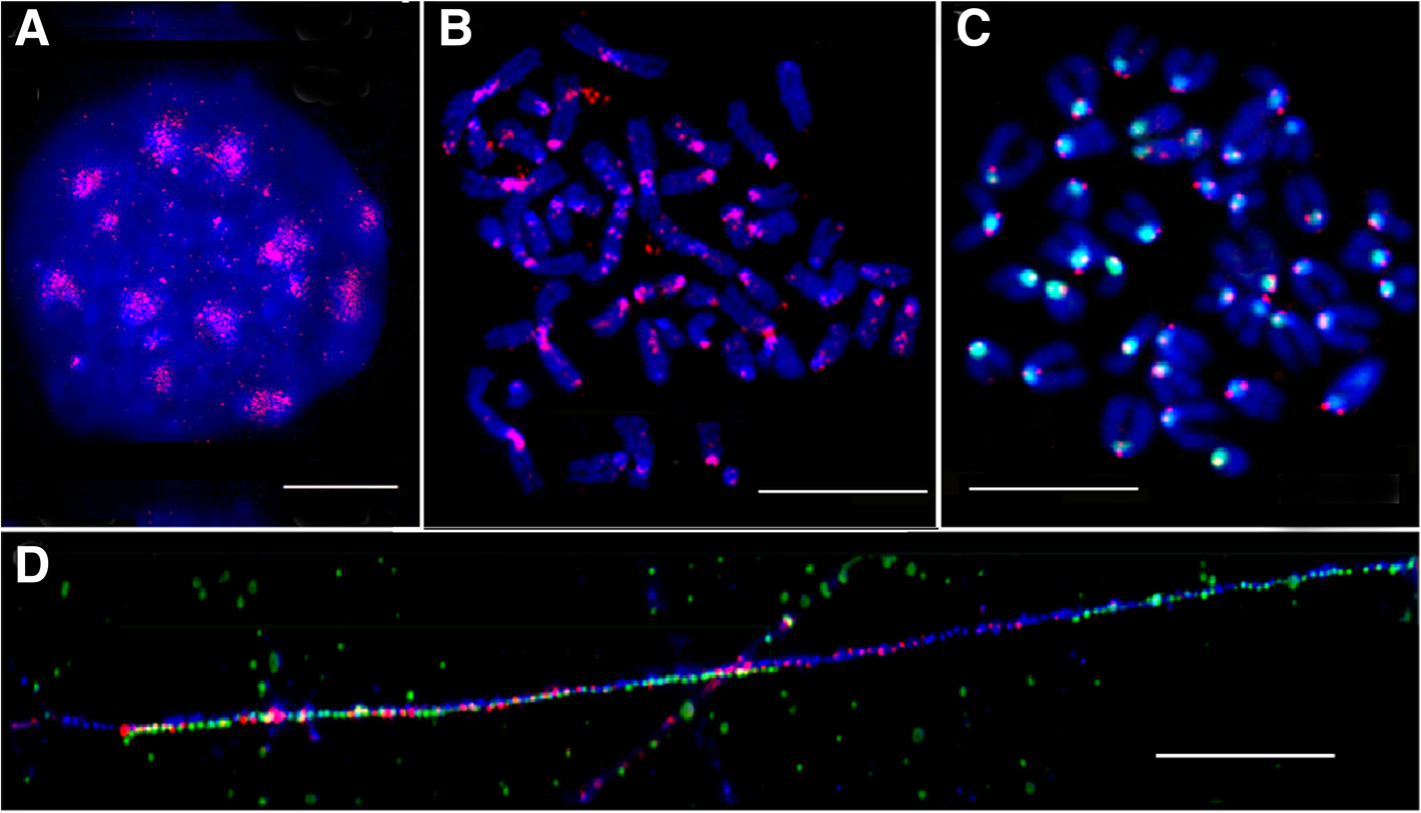
FISH and fiber-FISH with IAP probe on *M.musculus* nucleus, metaphase chromosomes and extended chromatin. **a** – IAP probe (red) on interphase nucleus from L929 cell culture; **b** - IAP probe (red) on metaphase plate from L929 cell culture; **c**– IAP probe (red) and MiSat (green) on bone marrow metaphase plate; **d** – fiber-FISH on L929 chromatin with IAP probe (red) and MiSat (green). DAPI counterstain is blue. Bar – 10 μm

The IAP probe enrichment is visible in the chromocenters (Fig. 5a). Both cell line L929 and bone marrow chromosomes are heavily labeled in centromeric regions. The ordinary FISH resolution on metaphase plates does not allow to determine the precise probe localization – whether it is centromeric or pericentromeric. The only reasonable conclusion is that the probe is localized close to the primary constriction. So, the IAP probe position is broadly centromeric. The feature of the - malignant and rearranged line L929 [44, 54] is the presence of long fused chromosomes with several heterochromatic blocks along the arms. These very blocks are also labeled and some staining could also be found in some subtelomeric regions (Fig. 5b). On metaphase plate from bone marrow with normal karyotype IAP strong signals were observed in centromeric regions of all chromosomes. Double FISH with MiSat makes us to suggest rather a periCEN location of IAP probe though some overlapping with MiSat occur (Fig. 5c). Double fiber-FISH on L929 chromatin was performed (Fig. 5d) which demonstrated that the IAP probe is located in the region at the border of MiSat array.

Thus, ERV and especially IAP definitely are the members of chromocenters. Our data evidenced for IAP as a regular member of heterochromatic periCEN.

### LINE in chromocenters

**Fig. 6:**
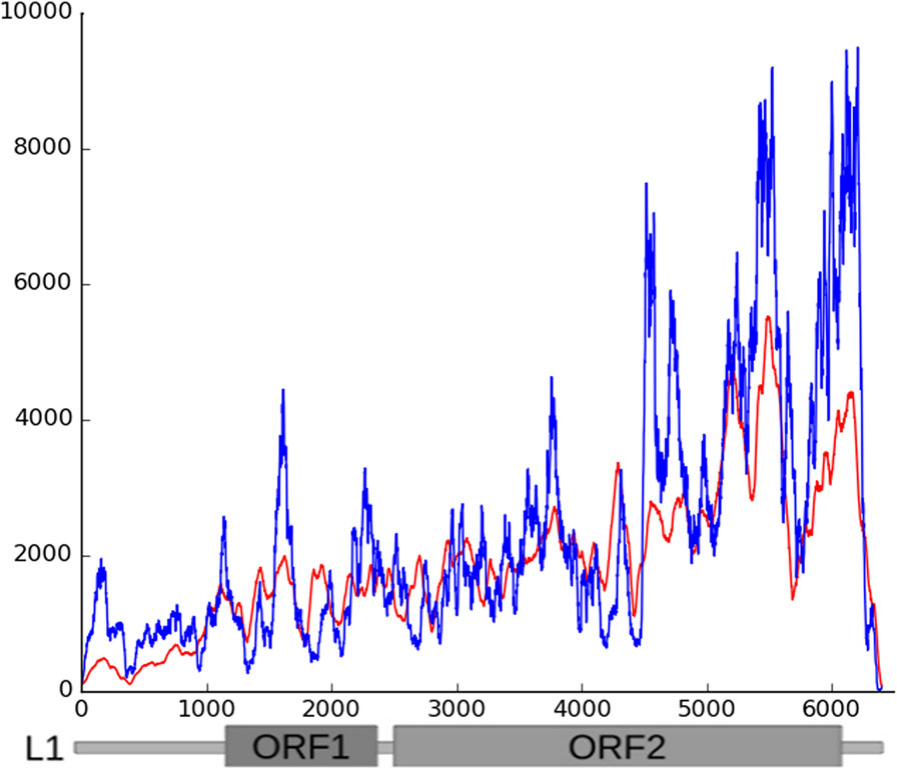
Reads coverage on mouse L1 consensus from Repbase. Blue line – reads from ChrmC dataset. Red line – reads from wgHTS on normalized on dataset size. X axis – LINE consensus indicated in bp.; Y axis – each nucleotide coverage

The coverage of the L1_MM consensus by reads to is far of being uniform (Fig. 6) in contrast to the same type of data for ERV (Fig. 3). Several peaks are visible on the graph and the majority of highest peaks are located in the ~ 2 kb area at the 3′ end of the second ORF and 3’ UTR (Fig. 6, blue line). The reads from wgHTS were also compared with the L1_MM consensus (Fig. 6, red line). The enrichment of peaks is visible in the same area with lower representation. So, the precise LINE fragment is a member of constitutive heterochromatin. The enrichment of this fragment in the whole genome data could be just due to the vast amount of heterochromatin.

## Discussion

### Overall chromocenter content

Chromocenters are well distinguished in mouse cell nuclei, they could be isolated biochemically and their DNA could be extracted in an amount sufficient for future sequencing without errors induced by amplification. The disadvantage is that the isolation method includes the step of mild ultrasonication, which breaks chromocenter DNA. The resulting reads are rather short, but these circumstances do not prevent chromocenter content determination. PacBio sequencing must facilitate the further full-scale assembly and the current paper provides reference points for the future assembly.

With all the limitations mentioned, we found unannotated dispersed sequences specific for sex chromosomes. Some of them (19 contigs, Table S1) were found only on Y chromosome and repeated there more than 500 times. The other 12 were found both in Y (500 times) and X (10–20 times) chromosomes (Table 3; Table S1). The heterochromatic nature of the sex chromosomes is well documented [6, 18, 55], though the detailed description of X/Y chromosomes repeats content is not done yet. Obviously, heterochromatic probes specific for both sex chromosomes or only for the Y chromosome could be designed on the base of the newly found fragments. These kind of probes will be very useful for revealing heterochromatic regions of murine sex chromosomes, which are not labeled by any commercially available chromosome-specific probes (routinely used in molecular cytogenetic studies) [35] and, consequently, the probes will provide a possibility to determine sex chromosome associations.

The position of the only gene fragment found in ChrmC dataset confirms the purity of chromocenter isolation, as the S*fi1* homolog gene is localized in the chromosome 11 periCEN region in the reference genome, i.e. very close to GPG.

### Tandem repeats (TR) in chromocenters

CEN/periCEN TRs change rapidly during evolution despite their conserved function at the centromeric locus [49, 56, 57]. In addition to extreme diversity in nucleotide sequences between species, CEN TR are typically characterized by sequential arrangement of monomers in the form of long arrays with the monomer length somehow corresponding to the size of nucleosomal DNA of ~ 170 bp [58].

The computational genome-wide TR analysis appears with the growing amount of the genomes sequenced [59, 60] and abundance and diversity of TR along evolutionary tree is striking. There are very few works with whole genome analysis of the most species TR sets (*Daphnia*, *Tribolium*, *Mus musculus*).

Our mouse large TR classification is based on sequence similarity, chromosome position, monomer length, monomers variability in the array, and the GC content; previously we identified four superfamilies, eight families, and 62 subfamilies - including 60 not previously described in spite of the extensive use of the laboratory mouse [34].

Some new TRs have already been tested in situ (Table 4, colored) and these TR are located in centromeric and/or subtrelomeric chromosome regions, i.e. regions of constitutive heterochromatin [34, 49] (for example see Additional file 3: Figure S1).

Surprisingly, only 33 out of 62 TR families defined in mouse WGS exist in ChrmC (Table 4) and together they comprise ~ 1% of ChrmC dataset. So, the rest of the TRs are presumably spread along the arms. As studies of the distribution of TR in mouse and human reference genomes were necessary, we did it taken into consideration the GC content of each TR. As a result a number of TRs have been found along chromosome arms [61]. TR arrays with euchromatic location were found in human [62, 63] and beetle [64]. Bioinformatics analysis of assembled *Tribolium castaneum* genome disclosed significant amount of TRs in euchromatic chromosomal arms and a clear predominance of satellite DNA-typical ~ 170 bp monomers in arrays of ≥5 repeats [64]. Comprehensive bioinformatics analysis of the large arrays of TRs (> 10 kb) located in the euchromatic part of the human genome showed a wide range of monomer size variations, from several nucleotides to several kilobases [63]. Thus, the existence of a number of TR distributed outside the centromeric regions, in euchromatic chromosomal arms, could be expected.

The TR intercalated along chromosome arms await for a special investigation. Their role could be quite important in organism morphogenesis. It is shown that variations in the number of repeats in the TR arrays placed near the dog developmental genes lead to the swift, yet topologically conservative morphological evolution of dog skulls [65]. From ChrmC data set analysis we can conclude that nearly all MaSat and MiSat arrays are included in chromocenters but TR families constitute only part of the remaining sequences (Table 4).

### Transposone related fragments in chromocenters

#### ERV

Endogenous retroviruses fall into three classes (ERV1–3), though with a markedly dissimilar evolutionary history in human and mouse. Notably, some ERVs are nearly extinct in human, whereas all three classes have active members in mouse [66].

ERVs by themselves represent ~ 10% of the mouse genome [52] and nearly the same figure is characteristic for the ChrmC dataset, but ERV representation in ChrmC differs from the reference genome. ERV3 class includes the non-autonomous MaLRs; with 388,000 recognizable copies in mouse, it is the single most successful LTR element. MTA belongs to the ERV3 class, MaLR-LTR family [53]. MaLR is still active in mouse and represented by MERV3, the MTA and ORR1 MaLRs [66]. MERV3 and MTA (ERV3 class) represent 2d and 3rd representative in ChrmC, but ERV2 class prevails.

Among active elements in mouse there are two abundant and active groups, the intracisternal-A particles (IAP) and the early-transposons (ETn). About 15% of all spontaneous mouse mutants have an allele associated with IAP or ETn insertion, demonstrating the functional consequences of ERV2 activity in mice [66]. Namely ERV2 class including IAP is the most abundant chromocenters’ ERV component (Tables 1, 5, Additional file 4: Table S2 and Additional file 5: Table S3).

Two families of the mouse TR with similarity to transposable elements (TE) were found during an intrinsic TR classification [34]. The array formed by these families has large monomers with a low degree of diversity and similar GC-content in both families. We have already found a TR class, TR-MTA, formed by MTA fragments. For TR-MTA family we found two loci with array length ~ 10 kb when mapped to the reference genome by BLAST under strict conditions [34]. The MTA based TR monomer includes the whole internal part of the element and an LTR [34], so MTA fragments in the ChrmC dataset could be expected (Fig. 4, MTA). MTA (ERV3 class) do present in ChrmC (Table 5) but not in the TR form.

It has been shown that distinct retroelement classes (TE) define evolutionary breakpoints demarcating sites of evolutionary novelty, namely LINEs and ERVs [67]. “Evolutionary breakpoints” of the mammalian genome are specific genomic locations that are “reused” during karyotypic evolution. When the phylogenetic trajectory of orthologous chromosome segments is considered, many of these evolutionary breakpoints are coincident with ancient centromere activity as well as a new CEN formation. Transcriptional units, comprised of satellites and a retrovirus, are bound by centromere proteins and represent a source of a novel small RNA class. The ERV, from which these small RNAs derived, is now known to be located in the centromere domain of several vertebrate classes. Discovery of this RNA form brings together several independent lines of evidence that point to a conserved retroviral-encoded processed RNA entity within eukaryotic CEN [68, 69].

LTRs of integrated retroviruses typically act as strong transcriptional promoters and in some cases promote transcription bidirectionally [70–72]. ERVs determined as an essential ChrmC component and especially their LTR may act as the transcriptional promoters for the surrounding TRs. Our results confirm the possibility of the ERV2 class (IAP) to underlie the evolutionary breakpoints in mouse periCEN regions.

#### Line

It has been shown cytologically that the major interspersed repeat families of the mouse, the LINE L1 element and the SINEs, occupy discrete positions on metaphase chromosomes, which correspond to G bands and R bands, respectively [73]. The probes routinely used are probes L1 (the major class of the long interspersed repetitive sequences, LINE; enriched in G bands) and B1 (the major class of the short interspersed repetitive sequences related to human Alu sequences, SINE; enriched in R bands) [74]. The G/R banding of chromosome arms is easy to obtain with L1/B1 double FISH while CEN/periCEN, i.e. constitutive heterochromatic regions, remain free from the label. Only MaSat can reveal C bands among the probes used [74]. The genomes sequencing confirms and underlines the most notable features about repeat elements: the contrast in genomic distribution of LINEs and SINEs. Whereas LINEs are strongly biased towards (A + T)-rich regions, SINEs are strongly biased towards (G + C)-rich regions [75]. Nowadays with ~ 2200 (~ 750 animals) of eukaryotic genomes available and the tendency of SINEs and LINE distribution confirmed for most of them, the mystery of this fact remains. If the LINEs and SINEs are retroposons that were dispersed through the genome by reintegration of reverse transcriptase products, why the heterochromatic TR rich regions depleted of them?

The presence of LINEs in centromeres was demonstrated using bioinformatics analysis of sequencing data in functional human centromere [76] and neocentromere [77], where full-length copies are taken into consideration. Still, the probes for L1 ORF did not paint the mouse heterochromatic regions [74]. The paradox could be solved with the assumption that not a full-length LINE but its fragments are the essential and numerous heterochromatic components.

In the process of classifying mouse large TR we already observed the L1 fragment-based TR [34]. L1 related TR included a part of the ORF2 and a 3′-end (3′ UTR) in their monomers. TE-related arrays were mapped to the reference genome in silico. Most of the loci found for the TR-L1 family did not exceed 5 kb. All loci were displayed on banded chromosomes of the reference genome. The facultative heterochromatin bands are definitely enriched in repeats from the TR-L1 family. Their increased concentration on the X chromosome was noted, but no TE related TRs were found on the Y chromosome [34]. So, the presence of the L1 part could be expected in the constitutive heterochromatin and it is confirmed by the present study (Table 1, Fig. 6), but not in TR form.

Experimental validation of these findings by FISH was performed by cloning, sequencing and mapping of DOP amplified ChrmC DNA [35]. The clones we selected for FISH, cover the whole ~ 2 kb fragment of L1 ORF2 and Lx. The same type of LINE fragments is found in assembly of human CENs [24]. The CEN assembly of human CENs is available in databases (LinearCen 1.1, http://www.ncbi.nlm.nih.gov/assembly/GCA_000442335.2). We mapped all the LINE fragments found in two human CENs to the RepBase LINE consensus and the concentration of fragments in the same region at the end of the second ORF is clearly visible [35]. We conclude that the precise LINE ~ 2-kb fragment, but not the full-length LINE, is the component of mouse and human constitutive heterochromatin enriched with TRs.

The current work made by a different method, i.e. with HTS, confirms our previous finding. Representation of reads recognized by Bowtie2 as LINE derived is no less than ~ 11% of the whole set (Table 1). Read coverage definitely shows an enrichment in the same fragment ~ 2-kb at the end of the L1 ORF2 (Fig. 6). The view of the whole coverage does not exclude the existence of full length LINE copies but in lesser amount than LINE fragment. Murine specific families LX and LX7 are also members of ChrmC dataset although they could not be shown due to the fact that their consensuses differ from the Repbase full-scale one (Fig. 6). They are truncated just up to the same fragment (LX and LX7 Repbase definition).

The same fragment type has been reported for the chicken periCEN region: 770 bp repeat based on a highly conserved 3′ region and a badly truncated 5′ end of CR1 element (LINE class) [78].

Human and mouse centromeres coalesce forming common clusters with human artificial chromosome (HAC), despite the fact that human α-satellite sequences and MiSat repeats lack homology and have only the 17 bp CENP-B box sequences in common [15, 79]. LINE fragment could be the certain genomic sequence responsible for heterochromatic regions recognition, being the most representative TE fragment both in mouse (Table 1) and human [35] CEN/periCEN regions.

The high amount of the precise LINE fragments in constitutive heterochromatin provides a solution for the FISH paradox: in situ hybridization could not recognize full scale LINEs in heterochromatin (there are very few of them) but the LINE fragment labels heterochromatin specifically [35].

### Perspectives

Although some specific components that tether heterochromatin within silencing regions have been identified, heterochromatin self-association is likely an additional driving force in the formation and maintenance of nuclei 3D. The tendency of repressed chromatin to cluster together [16–18] has suggested a birds-of-a-feather-flock-together model in which heterochromatin self-association drives global separation of the silencing compartment from the active, euchromatic compartment [80]. Still the model awaits for its validation. It is not known to what extent the particular organization of centromeres, telomeres, and repetitive sequences within the repressive compartment affect global nuclear organization. There are several areas where the knowledge is deficient with respect to heterochromatin formation and function. First and foremost, the annotation of repetitive DNA sequences is quite incomplete [6]. The amount of probes specific for the heterochromatic regions is quite limited. The current work is the first attempt to evaluate the chromocenters’ content and results of the work helps to fill up some of the gaps for the mouse.

## Conclusions

Among ChrmC MiSeq reads the most abundant are MaSat (66%) and MiSat (4%). The rest of TR (~ 1%) represent the TRs families previously described [34]. The rest of ChrmC dataset are mostly unannotated sequences, but some of them were identified when part of ChrmC dataset was assembled into contigs by IDBA_UD program. There are many fragments of Y chromosome, some rDNA and six other pseudo-genes and ncDNA gene identified in the assembled contigs. A fragment of gene ***sfi1*** homolog is found in contigs and localized to the chromosome 11 pericentromeric region. The ERV distribution differs from the whole genome: IAP (ERV2 class) is the most numerous. IAP and its LTR also prevail in the WGS dataset of TR containing contigs. Most of the LINE fragments come to the 2 kb region at the end of the 2nd ORF and its’ flanking region (3’UTR). The same region of LINE is the origin for the L1-based TR. Sequencing of chromocenters’ DNA (ChrmC) reveal IAP with LTR and precise LINE’ fragment of 2 kb as substantial mouse constitutive heteroсhromatin components together with TR.

## Additional files

Additional file 1: **Table S1.** Contig annotation. (XLS 13 kb)

Additional file 2: **Supplementary 1.** Syntethic IAP probe. (PDF 79 kb)

Additional file 3: **Figure S1.** Interphase and metaphase (31B) nuclei hybridized with the probes indicated on each panel. 84A and 31B - one probe FISH; 31A/31C – two-color FISH. Nuclei counterstained with DAPI (blue); the color of probes indicated. Scale bar 10 μm. (PDF 352 kb)

Additional file 4: **Table S2.** Contigs from mouse WGS with MiSat and ERV fragments. (XLS 31 kb)

Additional file 5: **Table S3.** Contigs from mouse WGS with TRPC-21A and ERV fragments. (XLS 32 kb)

## Abbreviations

BAC: Bacterial artificial chromosome
CEN: Centromeric
CEN/periCEN: Centromeric/pericentromeric
ChrmC: Reads collection of chromocenters’ DNA
ERV: Endogenous retroviruses
FISH: Fluorescent in situ hybridization
GPG: 3 Mb empty space reserved for centromere in most assembled genomes
HAC: Human artificial chromosome
HTS: High-Throughput sequencing
IAP: Inracisternal A-particle
LINE: Long interspersed nuclear element
LTR: Long terminal repeat
MaSat: Mouse major satellite repeat
MiSat: Mouse minor satellite repeat
ORF: Open reading frame
periCEN: Pericentromeric
SINE: Short interspersed nuclear element
TE: Transposable element
TR: Tandem repeats
UTR: Untranslated region
wgHTS: Whole genome high-throughput sequencing
WGS: Whole Genome sequencing
