# Supplementary material for: Mouse chromocenters DNA content: sequencing and in silico analysis
Source: BMC Genomics. 2018 Feb 20;19:151. doi: 10.1186/s12864-018-4534-z (PMC5819297; doi:10.1186/s12864-018-4534-z)
Supplement: Supplementary file 2 — Supplementary 1. Syntethic IAP probe. (PDF 79 kb) [file 12864_2018_4534_MOESM2_ESM.pdf]

Supplementary 1

Synthetic IAP probe of 196 bp was based on consensus sequence of IAP fragments from 5 mouse BAC-clone . IAP probe shows the similarity up to 95% with IAP in BAC clones.

5’-

ATCCTGGTTTTGGGACAAGTTAAGGTTCACAGTTTTGGGACAAGTTGCACCAAGCAC

CATTGGGACTTGAGGTTTGCAGAGGAACAGAGGGTTTTACTAAAAAGGGTTTTGCTG

GTCTGGAAGTTAGTGAGAAATTGCATAGAAGATAAAGATGTGGCACAGAAGTCCAGA

AAAGTAATGGATATCGTCGCTCCAG-3’

BAC	Chromosome	Distance from GPG, Mb
RP23-2K13	1	8.3
RP23-408K8	5	8.7
RP23-427J23	9	12.8
RP23-377C12	9	12.6
RP23-223E16	13	0.1

BAC – list of BACs used for the IAP synthetic probe construction; name of BAC clone and its chromosome location indicated.
