# Supplementary material for: Mouse chromocenters DNA content: sequencing and in silico analysis
Source: BMC Genomics. 2018 Feb 20;19:151. doi: 10.1186/s12864-018-4534-z (PMC5819297; doi:10.1186/s12864-018-4534-z)
Supplement: Supplementary file 3 — Figure S1. Interphase and metaphase (31B) nuclei hybridized with the probes indicated on each panel. 84A and 31B - one probe FISH; 31A/31C – two-color FISH. Nuclei counterstained with DAPI (blue); the color of probes indicated. Scale bar 10 μm. (PDF 352 kb) [file 12864_2018_4534_MOESM3_ESM.pdf]

Supplementary Figure S1

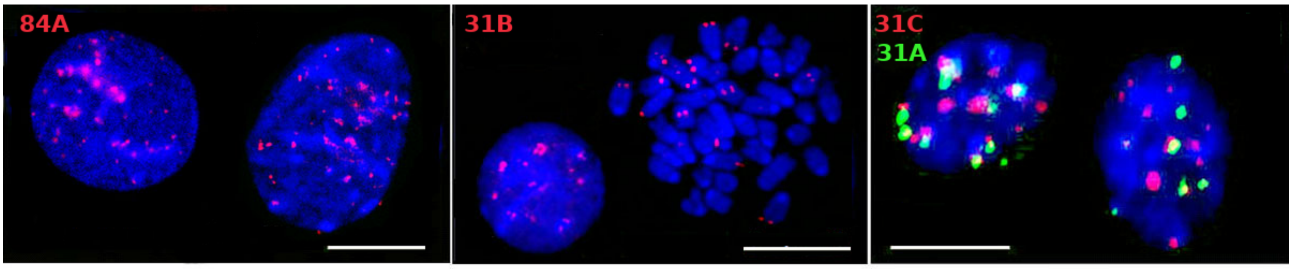

Figure S1. Interphase and metaphase (31B) nuclei hybridized with the probes indicated on each panel. 84A and 31B - one probe FISH; 31A/31C – two-color FISH. Nuclei counterstained with DAPI (blue); the color of probes indicated. Scale bar 10 µm.
